# Sex-based survival disparities persist in liver transplantation: MELD 3.0 fails to improve survival for waitlisted women

**DOI:** 10.3389/frtra.2026.1755115

**Published:** 2026-05-25

**Authors:** Marissa Di Napoli, Trevor Nydam, Michael Kriss, Maria Baimas-George, Bryon Bhagwandin, Bruce Kaplan, John Malamon

**Affiliations:** 1 Department of Surgery, Division of Transplant Surgery, Anschutz Medical Campus, University of Colorado, Aurora, CO, United States; 2 Colorado Center for Transplantation Care, Research and Education, Anschutz Medical Campus, University of Colorado, Aurora, CO, United States; 3 Department of Medicine, Division of Gastroenterology and Hepatology, Anschutz Medical Campus, University of Colorado, Aurora, CO, United States, United States; 4 Department of Medicine, Anschutz Medical Campus, University of Colorado, Aurora, CO, United States

**Keywords:** age, disparites, liver, MELD 3.0, sex, survival, transplant

## Abstract

**Background & aims:**

As a response to the well-documented disadvantages within the current allocation system, MELD 3.0 emerged on July 15, 2023, to more accurately characterize end-stage liver disease severity across the sexes. While simulation modeling predicted fewer waitlist deaths compared to MELD-Na, there is a lack of empirical data evaluating the efficacy of MELD 3.0 since its implementation. This study aims to conduct a waitlist patient survival study to explore sex- and age-based survival disparities in women and quantify empirical survival differences among waitlisted liver transplant candidates before and after the implementation of MELD 3.0.

**Methods:**

A retrospective, cross-sectional patient risk analysis of the Scientific Registry of Transplant Recipients (SRTR) database of liver transplant candidates listed from January 1, 2017, to September 3, 2024.

**Setting:**

The United States.

**Participants:**

Adult liver transplant candidates on the waitlist (*N* = 95, 211). Exclusion criteria included a history of prior transplant and the need for multi-organ transplant.

**Exposures:**

All-cause patient waitlist mortality.

**Main outcomes and measures:**

The primary outcome was the persistence of the waitlist survival gap between the sexes before and after the implementation of MELD 3.0. Secondary outcomes included risk-adjusted survival disparities in association with age.

**Results:**

Across the two MELD eras, waitlisted females experienced a 16.28% mean increase in the relative 180-day median hazard ratio as compared to males. Patients 65 years and older had a 350% mean increase in their 180-day mortality hazard ratio as compared to patients 18 to 35 years of age. Patients 65 years of age and older with MELD scores greater than 34 experienced a 180-day median survival probability of 65%.

**Conclusions and relevance:**

Despite the implementation of MELD 3.0, the waitlist survival gap disadvantaging women persists. The relationships between waitlist survival and patient age and sex are complex and time-dependent. As such, time-dependent survival models along with the addition of patient age and other characteristics could significantly improve patient survival diagnostics and help close the waitlist survival gap between the sexes.

## 1 Introduction

In 2002, the Model for End-stage Liver Disease (MELD) score was adopted by the United Network for Organ Sharing (UNOS) in an effort to prioritize the most ill or high-risk patients for organ allocation on the liver transplant waitlist (1, 2). With a goal to rank waitlisted patients in order of exigency, this prognostic score supplanted subjective descriptors with an objective calculation of liver disease severity to predict three-month patient survival using commonly available laboratory tests (3). Since its adoption, the MELD score has been revised twice to refine and improve its predictive accuracy and thus, more effectively prioritize patients for liver allocation. For instance, in 2016, a new version (MELD-Na) emerged with the addition of serum sodium, an independent predictor of mortality for waitlisted patients, which successfully lowered waitlist patient mortality by 27% without affecting post-transplant survival (4).

While the implementation of the standardized MELD system was successful in decreasing waitlist mortality, there remains significant room for improvement given the persistence of age- and sex-related disparities (5). With continued improvements in medical management and bridging procedures, older patients are being approved for liver transplants, yet significant variations in the maximum patient age between centers exist given a lack of a universal consensus and scarce literature defining age-based waitlist mortality (6). On the other hand, sex-based disparities are well studied, with a well-defined female survival disadvantage splayed throughout MELD-era literature. When compared with men, women are less likely to undergo liver transplants and are more likely to die on the waitlist due primarily to body size and the overestimation of renal function (7–13). Further, women are less likely to receive MELD exception points given the incidence, such as hepatocellular carcinoma (HCC), is higher in men (14).

As a response to the sex-based disadvantages observed with MELD-Na, MELD 3.0 emerged in 2023 to more accurately characterize disease severity in women (15–17). MELD 3.0 was the first systematic intervention in over twenty years focused on addressing this gap in access to liver transplantation. The new revision incorporates serum albumin and recipient sex, modifies interactions between patient variables, and reduces the maximum serum creatinine value to 3.0 mg/dL. While simulation modeling predicted fewer waitlist deaths compared to MELD-Na, there remains a lack of empirical data and research evaluating the effect of MELD 3.0 on waitlist mortality and sex- and age-related disparities since its implementation as the current allocation system.

Given a steady increase in the average age of liver transplant patients (18), the transplantation community must find a way to balance efficacy and equitable organ access with regard to treating and caring for older waitlisted patients. We hypothesized that age is a significant survival factor and the MELD score underestimates the relative mortality risk of older patients. Because age is a known risk factor, we contend that not including age as a risk component is both unethical and misleading because older liver transplant patients do not receive additional MELD points for age, as their age is not included in any MELD calculation. All patients, independent of age, have the rights to privacy, informed consent, medical education, and the right to receive all relevant information regarding clinical practices and principles (19–21). Finally, we hypothesized that older female patients may experience increased risk as compared to younger patients and that age- and sex-related factors may interact to further disadvantage older female liver transplant patients.

The goal of this study was to evaluate the relationship between liver transplant waitlist mortality and age- and sex-based disparities using survival analysis across distinct MELD eras, or iterations. The Cox proportional-hazards model was selected for this study given it is a superior methodology in estimating patient survival when compared to logistic regression, which is the current diagnostic benchmark for end-stage liver disease (22). For example, Cox regression utilizes more patient information (censoring days), is more robust to the effects of covariates that are correlated, and offers a more interpretable and therefore more meaningful result (e.g., median survival days vs. the odds ratio of surviving a given number of days) (23). Finally, this is the first study to examine the impact of MELD 3.0 on the waitlist survival gap between the sexes.

## 2 Methods and materials

### 2.1 Data sources

Data from the Scientific Registry of Transplant Recipients (SRTR) was utilized. The SRTR dataset includes information on all donor, waitlisted candidates, and transplant recipients in the United States as submitted by members of the Organ Procurement and Transplantation Network (OPTN). The Health Resources and Services Administration (HRSA), U.S. Department of Health and Human Services provides oversight to the activities of the OPTN and SRTR contractors. The data reported here have been supplied by the Hennepin Healthcare Research Institute (HHRI) as the contractor for the Scientific Registry of Transplant Recipients (SRTR). The interpretation and reporting of these data are the responsibility of the authors and in no way should be seen as an official policy of or interpretation by the SRTR or the U.S. Government.

### 2.2 Study population

All single-organ adult liver transplant patients with no prior organ transplants who were listed between January 1, 2017, and September 3, 2024, were included (*N* = 95,211). Patients were excluded if they were listed for re-transplantation, multi-organ transplant, or had a prior transplant. There were 950 living donor transplants performed.

### 2.3 Statistical approach

Patient characteristics were described as means (standard deviation, SD) or medians for continuous variables. Outcomes, defined as categorical variables, were reported as the percentage within the group. For continuous variables, a two-way ANOVA test was used to measure the observed differences in patient characteristics and the independent variables used in this study. The chi-squared test was used to measure the significance of categorical and indicator (binary) variables. To provide an adequate number of participants per risk stratum and a consistent representation of patient mortality risk, study participants were stratified into six groups by their most recently captured MELD scores: 1) 6-14, 2) 15-19, 3) 20-24, 4) 25-29, 5) 30-34, and 6) 35-40. Biochemical MELD-Na and MELD 3.0 were utilized at the time nearest to censoring for all analyses. Patients were also stratified into four age groups at the time of listing: 1) 18-35 years; 2) 36-55 years; 3) 56-64 years; and 4) 65 years and greater (65>). The number of days survived was calculated as either the number of days from 1) listing to death, 2) listing to removal from the waitlist, or 3) listing to 180 days (180D) post-listing. Patients removed from the waitlist were censored on the day of removal from the waitlist. 180D follow-up periods were maintained and censored accordingly for all study cohorts. The last administrative follow-up was March 3, 2025, providing 180 days of follow-up for all study participants used in this study. Hazard ratios were calculated using unadjusted Cox proportional-hazards regression and by adjusting for age at listing, sex, race, and the most recent MELD score. Survival probability curves and at-risk tables were generated using the semiparametric Kaplan–Meier estimation (24). We tested the proportionality assumption for all models using the Schoenfeld residuals method (25). Mortality rates were calculated by dividing the number of patient deaths by the number of patients at risk produced by each Kaplan–Meier model at the 180D censoring period.

Two cohorts with equal listing days were created to compare patient risk and survival curves between the distinct MELD-Na and MELD 3.0 eras: 1) patients listed from the implementation of MELD 3.0 on July 15, 2023, to September 3, 2024 (443 listing days; *N* = 15,619), and 2) patients listed from August 25, 2022, to July 14, 2023 (443 listing days; *N* = 11,463). Both cohorts allowed for a full 180D follow-up period where no new patients were added to the respective cohorts. All analyses were performed using the R statistical language (version 4.5.0) (26). Throughout this study, we adhered to the Strengthening the Reporting of Observational Studies in Epidemiology reporting guidelines (27).

## 3 Results

### 3.1 Population Characteristics

A diagram of the exclusion criteria for this population was provided in Supplementary Figure 1. Across the entire population, waitlisted patients were 55.21 (SD = 11.44) years of age on average at the time of listing and most often male (61.65%) and Caucasian (86.94%). Only 6.67% of this population reported their race as African American. At the time of censoring, the most recent mean MELD score was 20.95 (SD = 10.29). MELD scores ranged from 6 to 40, as OPTN puts a cap on MELD scores greater than 40. Most waitlisted patients were educated up to or beyond high school (Table 1).

**Table 1:** Patient characteristics of liver transplant waitlist candidates, 2017-2024.

WAITLISTED PATIENTS (*N* = 95, 211)	
Characteristics	Value
AGE	
Mean (SD)	55.21 (11.44)
Median [Min, Max]	58.0 [18.0, 86.0]
Missing	0 (0%)
SEX	
Female	36, 514 (38.35%)
Male	58, 697 (61.65%)
Missing	0 (0%)
RACE	
Caucasian or Caucasian American	82, 777 (86.94%)
Black or African American	6, 352 (6.67%)
Asian or Asian American	3, 856 (4.05%)
Other	2, 226 (2.34%)
Missing	0 (0%)
EDUCATION	
Associate/bachelor's degree	20, 088 (20.1%)
Attended College/Technical School	23, 989 (25.2%)
Grade School (0-8)	5, 110 (5.37%)
High School (9-12)	35, 144 (36.92%)
None	443 (0.47%)
Post-College Graduate Degree	7, 662 (8.05%)
Unknown	2,765 (2.9%)
MELD	
Mean (SD)	20.95 (10.29)
Median [Min, Max]	19 [6, 82]
Missing	0 (0%)

### 3.2 Age-stratified survival disparities

**Figure 1:**
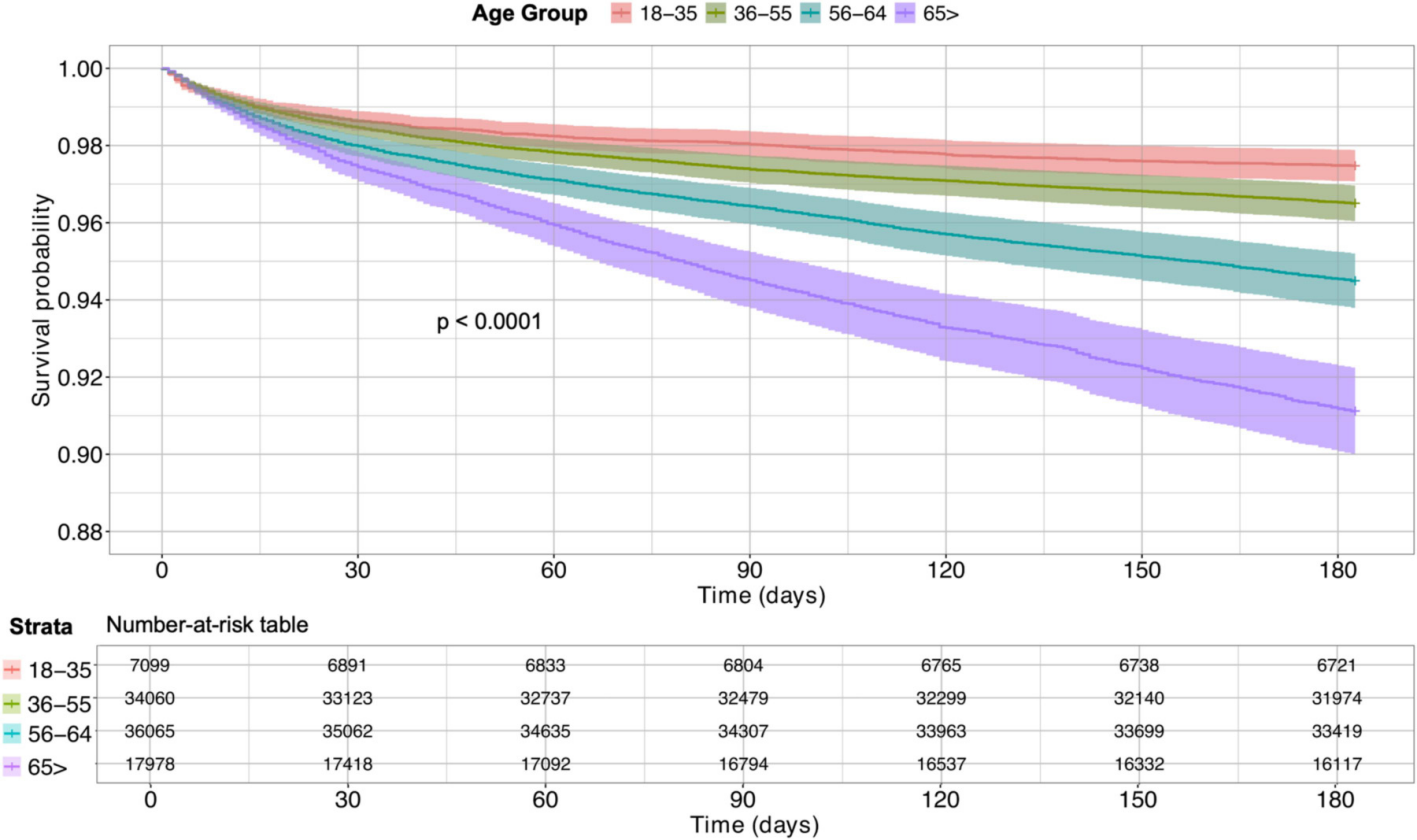
**Risk-adjusted and age-stratified survival disparities.** Patients were stratified into four age groups. Covariate-adjusted 180-day survival probability curves and median survival days were calculated for each age stratum using the Cox proportional-hazard regression and Kaplan–Meier estimation. The Kaplan–Meier estimations were adjusted for MELD score, sex, and race.

**Figure 2:**
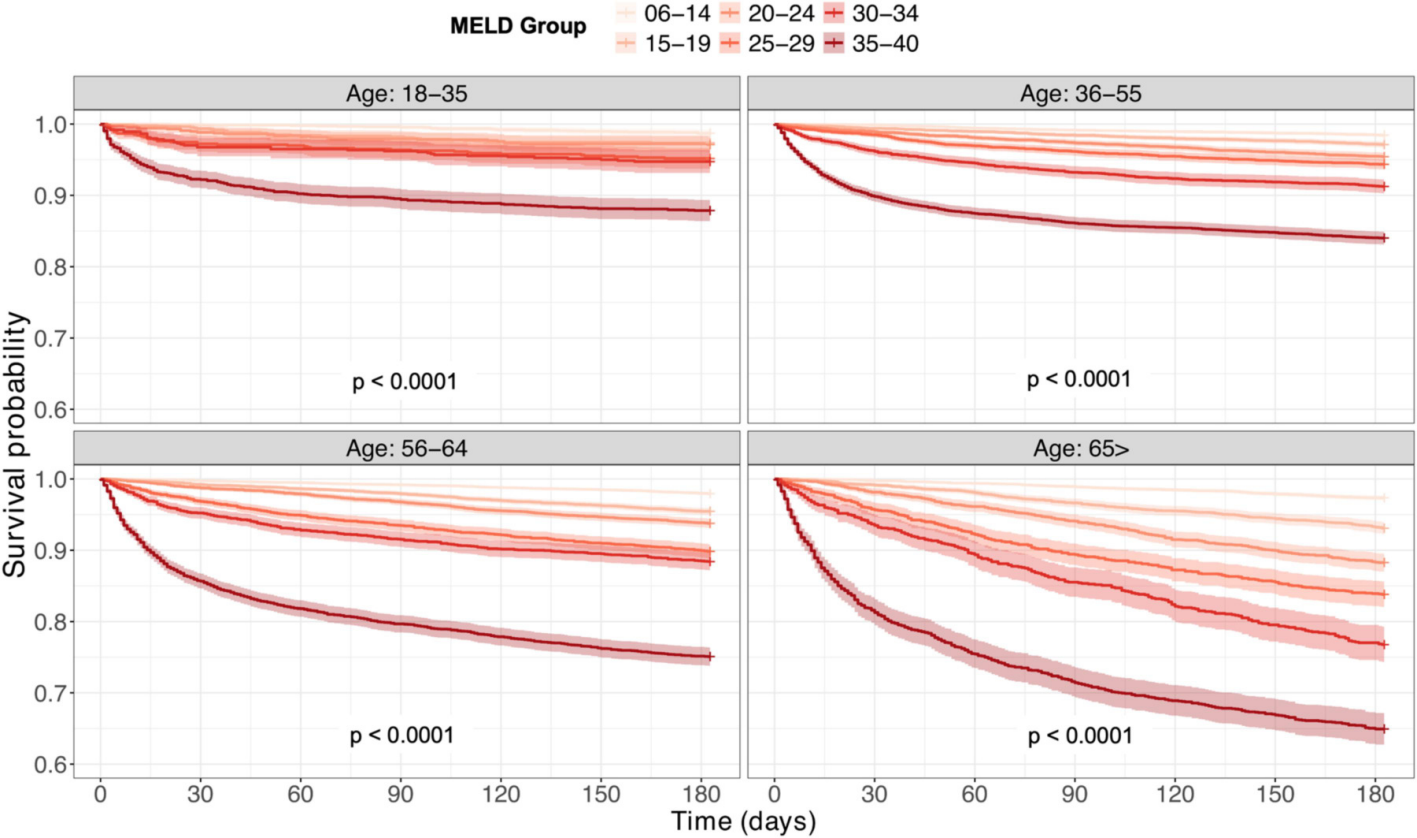
**Risk-stratified survival disparities by age group.** Patients were stratified into four age groups and six MELD groups. Covariate-adjusted 180-day survival probability curves and median survival days were calculated for each stratum using the Cox proportional-hazard regression and Kaplan–Meier estimation. The Kaplan–Meier curves were adjusted for sex and race. Each survival curve was faceted by the age group.

All models presented herein passed the Schoenfeld residuals tests for proportionality with *p*-values greater than 0.2. Age stratification revealed highly significant differences in 180D median patient survival (*p* < 0.0001, Figures 1, 2), where survival probability steadily decreased with patient age. The 18 to 35 age group had a mortality rate of 5.34% (379/7099) and a median 180D survival probability of 97.47% (97.07, 97.88, SE = 0.0021, Supplementary Table 1). The 36 to 55 age group had a 6.19% mortality rate and a 180D median survival probability of 96.55% (96.07, 96.99, SE = 0.0017). The 56 to 64 age group had an adjusted mortality rate of 7.41% and a median adjusted 180D survival probability of 94.54% (93.85, 95.24, SE = 0.0012). The 65 > age group had a 10.46% mortality and a mean adjusted 180D survival probability of 91.18% (90.08, 92.29, SE = 0.0062). As compared to the 18 to 35 age group, patients 65 and older had a 180D hazard ratio of 3.5 (CI = 3.16, 3.95, *p* < 0.001, Supplementary Figure 2).

### 3.3 Age-related disparities between sexes

**Figure 3:**
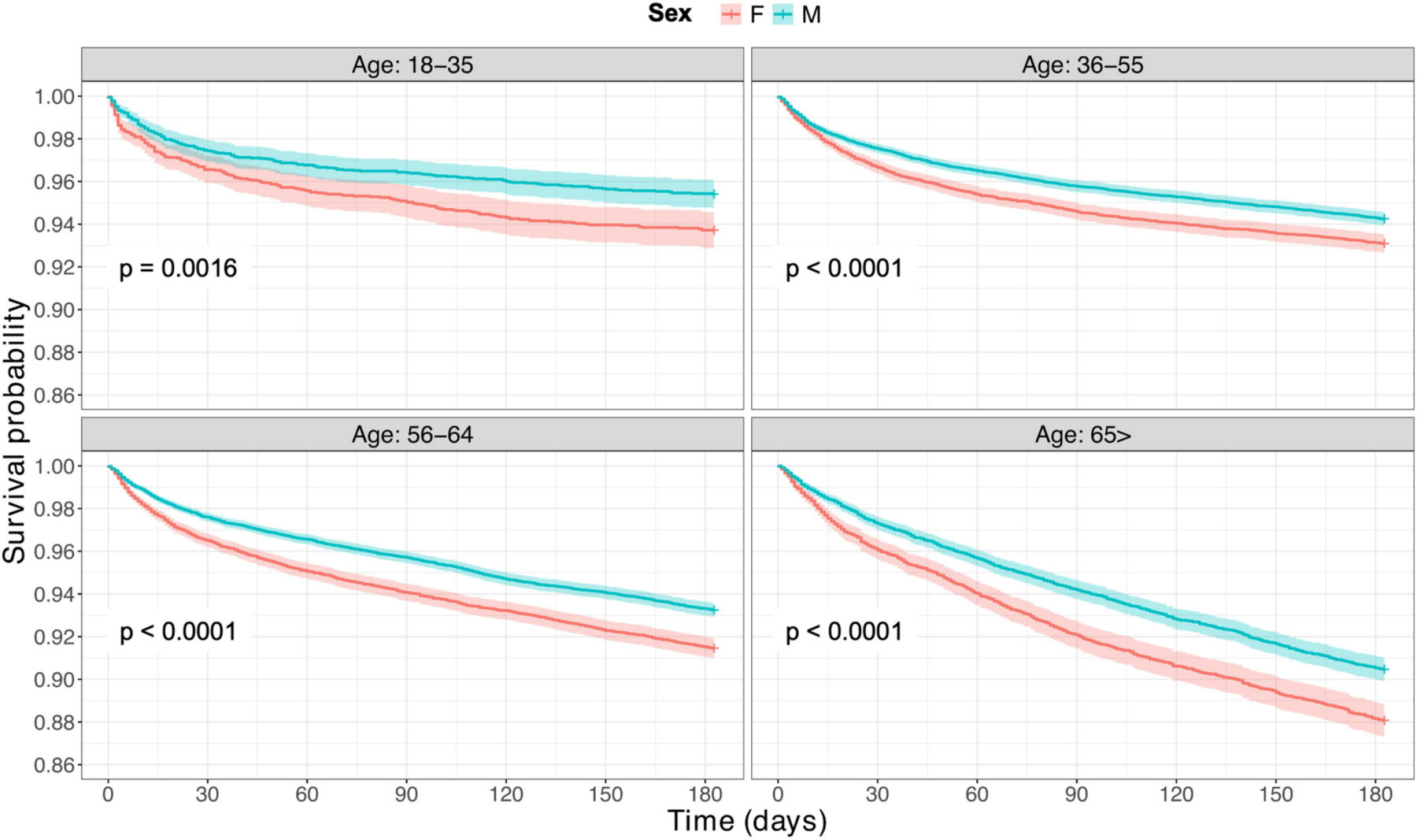
**Risk-adjusted survival disparities stratified by sex and age.** Patients were stratified by sex and age. Covariate-adjusted 180-day survival probability curves were calculated for each stratum using the Cox proportional-hazard regression and Kaplan–Meier estimation. The Kaplan–Meier curves were adjusted for MELD score and race. Each survival curve was faceted by the age group.

Overall, male patients had a highly significant reduction in the unadjusted and adjusted 180-day HR (0.8 and 0.9, Supplementary Figure 2). Sex-related survival differences were observed among age strata, with significant disparities observed in all four age groups. Waitlisted male patients aged 18 years and older had a significant 180D survival advantage (*p* < 0.0001, Figure 3). Female patients 65 years of age and older had a 180D median survival probability of 88.15% (CI = 87.39, 88.91, SE = 0.0044), while men had a survival probability of 90.53% (CI = 89.99, 91.08, SE = 0.0031, Supplementary Table 2), representing a 2.38% average relative decrease in the median 180D survival probability for women aged 65 and greater as compared to men in the same age category.

### 3.4 Sex-related disparities before and after MELD 3.0

**Figure 4:**
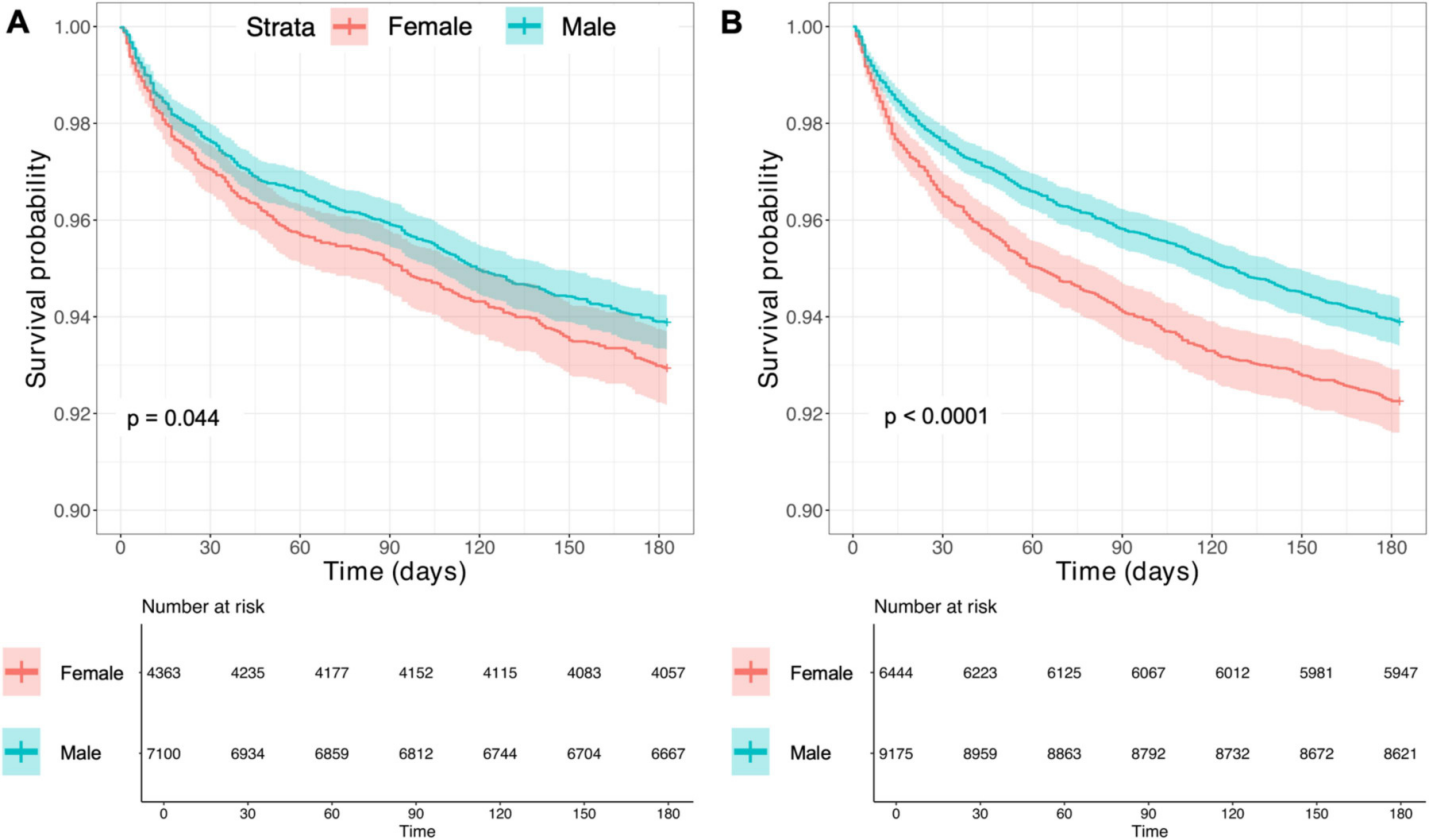
**Sex-related survival disparities in the MELD-Na and MELD 3.0 eras.** We created two cohorts: **(A)** a cohort consisting of 443 listing days from August 25, 2022, to July 14, 2023 (*N* = 11,463); **(B)** from the implementation of MELD 3.0 on July 15, 2023, to September 3, 2024, consisting of 443 days of patient listings (*N* = 15,619). 180-day survival probability curves were created using the Kaplan–Meier estimation.

Within the MELD-Na era, there was a mean 180D median survival probability of 92.94% (CI: 92.18, 93.7, SE = 0.0042, Figure 4) and 93.89% (CI = 93.33, 94.45, SE: 0.003, *p* = 0.044) for females and males, respectively. Since the implementation of MELD 3.0, there was a median 180D survival probability of 92.26% (CI = 91.61, 92.91, SE = 0.0036) and 92.77% (CI = 91.79, 93.76, SE = 0.0044, *p* < 0.0001) for females and males, respectively. Noticing an overall decrease in waitlist survival across these two distinct MELD eras, we performed Cox proportional-hazards regression to compare the 180D waitlist survival risk between these two eras. Both age and sex were highly significant (*p* < 0.0001) predictors of patient risk and survival. Survival analysis revealed no significant improvement in the waitlist survival gap between sexes and a total reduction in sex-related and aggregate patient survival between these two MELD eras. Finally, we examined population changes across the two distinct MELD eras, MELD-Na and MELD 3.0 (Supplementary Table 4). Although there was a slight increase (∼2%) in the total number of waitlisted female, the median patient height decreased two 2 centimeters on average. No secular changes in patient diagnoses were found. There was no difference in MELD exception points across the two eras.

## 4 Discussion

When UNOS implemented MELD 3.0 in July 2023, their overarching goal was to more accurately predict patient mortality as well as improve upon the current MELD-Na score by addressing sex-based disparities that disadvantaged women. Given its importance and the resources expended, there is relatively little research evaluating its success. To our knowledge, this study marks the first risk-stratified survival analysis of the efficacy of the MELD 3.0 allocation system. Importantly, waitlist survival differences provide an opportunity to continue to improve patient outcomes and patient risk models. The intended improvements to prognostics with the implementation of MELD 3.0 were primarily focused on enhancing the accuracy of assessing disease severity in women. MELD-Na underestimates renal function in women because women have a lower glomerular filtration rate (GFR) for the same creatinine value as men, which in turn leads to underestimation of mortality risk by up to 2.4 points (12). Changes to the MELD formula included the incorporation of female sex and serum albumin, modification of variable formula weights, introduction of interactions between bilirubin and sodium and between albumin and creatinine, and restricting the upper limit for creatinine to 3.0 mg/dL (17).

In the new MELD 3.0 era, this study found that both men and women had a decreased mean 180D survival probability when compared to the MELD-Na era. Using Cox regression and Kaplan–Meier analysis, we found a 16.28% relative mean increase in the 180-day hazard ratio and a 2.38% decrease in the median 180D survival probability of women waitlisted for liver transplant. These findings denote a failure of MELD 3.0 in effectively addressing and closing the waitlist survival gap between sexes. Interestingly, our findings directly contradict Kim *et al*., which led to the adoption of MELD 3.0. Their work utilized a Liver Simulation Allocation Model (LSAM), which predicted fewer waitlist deaths with MELD 3.0 versus MELD-Na (7,788 vs 7,850, respectively; *p* = 0.02). Additionally, they reported that 14.9% of women were recategorized to a higher MELD score with the new scoring system, suggesting a higher likelihood of graft allocation and thus, a lower risk of waitlist death (17). Given that Kim's work is based on a simulation model, it lacks realism and has significant technical limitations. Simulation models must be sufficiently calibrated and frequently readjusted to provide accurate and reliable estimations of population risk trends, a previously expressed concern given that the MELD 3.0 model only reported a concordance statistic (c-index) (28). LSAMs are not peer-reviewed or retrospectively validated. In light of this, our study, which evaluates actual patient outcomes over the course of 443 days, must be taken into consideration.

While our results, which demonstrate that MELD 3.0 did not improve the female waitlist survival gap, are somewhat difficult to directly explain, there are several known anthropomorphic and physiological factors that may not be accounted for by the MELD score alone. It has been documented that disparities in waitlist survival between sexes are likely multifactorial and cannot be explained by MELD score alone (29, 30). For example, MELD 3.0 capped serum creatinine at 3.0 mg/dL, lowering the impact of creatinine in the calculation, which only decreases the maximum component score by 1 point (17). Interestingly, other studies have succeeded in capturing more accurate mortality predictions with the replacement of creatinine by eGFR, given that it more accurately represents renal function (31, 32). Perhaps, further prognostic iterations should consider incorporation of eGFR or decreasing the weight of creatinine in the equation.

Prior findings have substantiated that women whose height is less than 166 centimeters experience higher rates of waitlist mortality (33). The addition of one or two MELD points to the shortest 8% of waitlisted female candidates would be sufficient in reducing waitlist mortality (34). However, during the development of MELD 3.0, Kim et al. believed that sex had a larger and more consistent effect than height and therefore selected to add points for only sex in the MELD 3.0 calculation (35). As such, MELD 3.0 adds 1.33 points for all female patients, regardless of height. While it seems logical that the addition of universal points for women may address differences in waitlist survival between sexes, it is still insufficient. This model does not correct for other factors that affect most women, such as a size mismatch with the average deceased liver donor or a lack of exception points. Consideration of these additional factors may account for the failure to improve female sex waitlist mortality in our results. And while optimization of the MELD score is clearly an important component to addressing disparities in waitlist survival, perhaps changes to allocation policies are also needed to address factors not accounted for by MELD 3.0 alone.

Within the highest-risk group (MELD >34), patients 65 years of age and older had a considerably lower 180D median survival probability as compared to patients 18 to 35 years of age (65% vs. 87.5%). This strong correlation between patient age and mortality risk was true in both men and women, but much more pronounced in women. We have cited ethical standards with regard to age to further emphasize the need to include age as a risk factor for waitlisted liver transplant patients. However, there is a simpler, experiential truth to this finding. Given equal MELD scores, if you were 65 years old, would you prefer to have the same allocation risk score as a 30-year-old patient? It is also critical to realize that MELD scores greater than 34 were associated with significantly worse and non-linear survival probabilities. Although it is possible to calculate a higher MELD score, in the current allocation system, MELD scores are functionally capped at 40, disadvantaging the sickest of patients. Given that waitlist mortality increases in a nonlinear fashion beyond a score of 40 and that outcomes are preserved and not inferior in this population, eliminating the cap on the MELD score may also improve survival disparities (35).

Our results highlight a complex interaction between age, sex, and waitlist survival. In future work, we plan to further evaluate the waitlist survival gap between sexes by analyzing relationships with liver disease etiology. We aim to perform risk stratification for other attributes to define additional risk factors that may be contributing to the survival disparities that female liver transplant candidates experience. The current allocation system relies on accurate predictions of waitlist mortality to ensure that the sickest patients receive the highest priority. Thus, it is crucial that efforts are made to optimize the prognostic value of the MELD score and minimize disparities. In summary, MELD 3.0 does not sufficiently address waitlist survival disparities at this juncture, continuing to disadvantage female liver transplant candidates, especially those older than 35 years of age.

## 5 Limitations

This study has limitations. First, this was a retrospective cross-sectional study using SRTR data, and as such, there is the potential for hidden selection bias. Additionally, this study included less than one year of waitlist registry data for evaluating MELD 3.0; however, the sample size for this cohort was sufficiently large (*N* = 15,619) and we do not believe that this impacted the significance of our results or the validity of our findings. Furthermore, this sample size allowed for the model to be tested to account for potential confounding variables, including age, ethnicity, and sex, to limit potential selection biases. Future studies will include additional participants. Upon comparing the MELD-Na to the MELD 3.0 cohort, we observed a median aggregate decrease of 2 centimeters in the population height. Further analyses will be needed to determine causality. Finally, we did not observe differences in the exception points given across the two MELD eras; nonetheless, further studies should stratify participants based on these exception points to further adjudicate their effects on waitlist survival. However, this will be challenging given that MELD 3.0 gives 1.33 additional points to women independent of their height.

## 6 Conclusions

Despite the efforts of MELD 3.0 to account for female sex disparities, it fails to improve the sex-based disparity in waitlist survival. Furthermore, our results demonstrate that the waitlist survival gap in female patients steadily increases with age, signifying interactions between aging, sex, and waitlist survival. When compared to the MELD score alone, survival models may help to more accurately gauge and address these disparities. We also recognize that true equity in liver transplantation may not be achieved without policy changes that account for the factors that disadvantage women beyond the MELD score. The liver transplantation community should consider possible measures to achieve equity in waitlist survival and access to liver transplantation, including ongoing efforts to optimize the MELD score to accurately reflect disease severity, the consideration of survival models, and modifications to current allocation policies.

## Data Availability

Publicly available datasets were analyzed in this study. This data can be found here: https://www.srtr.org/about-the-data/the-srtr-database/
