# Supplementary material for: Sex-based survival disparities persist in liver transplantation: MELD 3.0 fails to improve survival for waitlisted women
Source: Front Transplant. 2026 May 25;5:1755115. doi: 10.3389/frtra.2026.1755115 (PMC13243226; doi:10.3389/frtra.2026.1755115)
Supplement: Supplementary file 1 [file Table1.docx]

**Supplementary Appendix**

Supplement to: Age and *sex-based survival disparities persist in liver transplantation: MELD 3.0 fails to improve survival for waitlisted women.*
*The persistence of age- and sex-based survival disparities on the liver transplant waitlist.*

**Supplementary appendix for:**

*The persistence of age- and sex-based survival disparities on the liver transplant waitlist.*

**Contents**

**Supplemental Figure 1**. Participant workflow diagram. 3

**Supplemental Figure 2.** Forest plot of age-stratified and risk-adjusted 180-day patient mortality risk. 4

**Supplemental Table 1**. 180-day risk tables for risk-adjusted age stratified survival model 5

**Supplemental Table 2**. 180-day risk tables for sex and age stratified risk-adjusted survival model. 6

**Supplemental Table 3.** 180-day risk tables for pre- and post-MELD 3.0 groups stratified by sex 7

**Supplemental Table 4.** Population differences across the two distinct MELD eras 8

**Supplemental Figure 1.** Participant workflow diagram. The diagram below provides all inclusion criteria, and the participants included in this study.


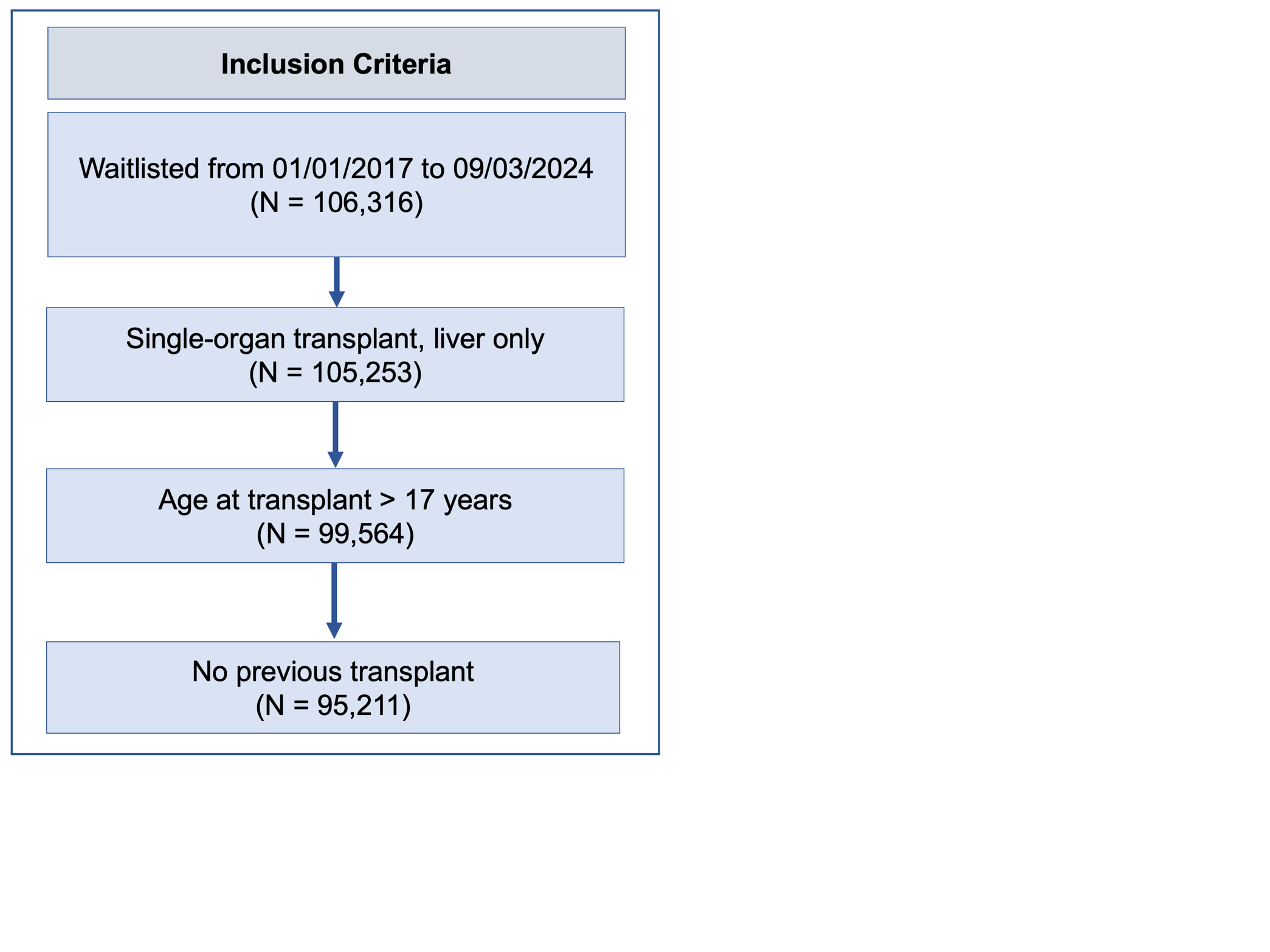


**Supplemental Figure 2.** Forest plot of age-stratified and risk-adjusted 180-day patient mortality risk

**
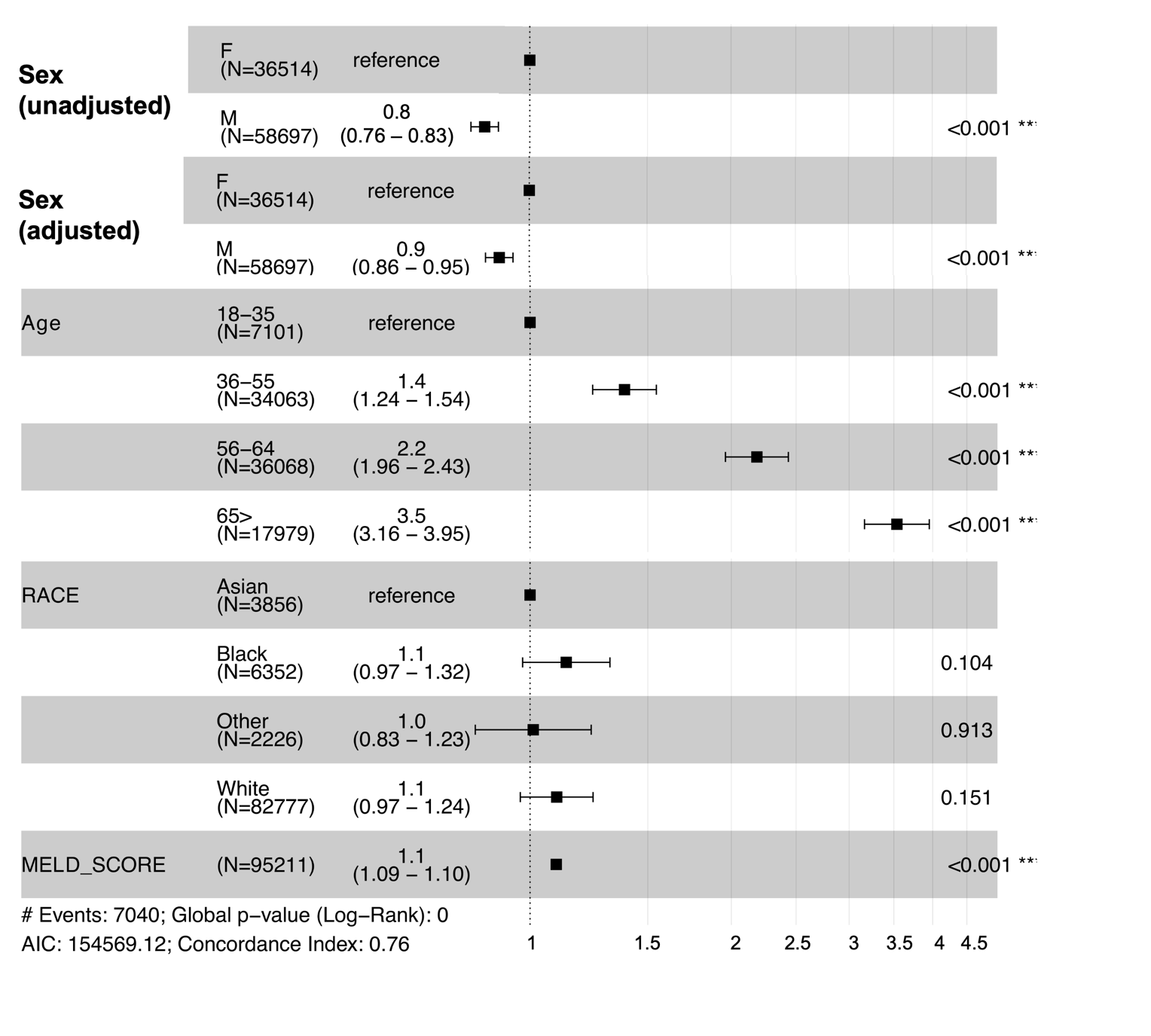
**

**Supplemental Table 1.** 180-day risk tables for risk-adjusted age stratified survival model.

| Strata | N | Number at Risk | Number of Events | Median Survival Probability | Lower 95% | Upper 95% | Standard Error |
| --- | --- | --- | --- | --- | --- | --- | --- |
| 18-35 | 7099 | 6721 | 379 | 0.9747 | 0.9707 | 0.9788 | 0.0021 |
| 36-55 | 34060 | 31974 | 2107 | 0.9653 | 0.9607 | 0.9699 | 0.0017 |
| 56-64 | 36065 | 33419 | 2674 | 0.9454 | 0.9385 | 0.9524 | 0.0012 |
| 65> | 17978 | 16117 | 1880 | 0.9118 | 0.9008 | 0.9229 | 0.0062 |

**Supplemental Table 2.** 180-day risk tables for sex and age stratified risk-adjusted survival model.

| Strata | N | Number at Risk | Number of Events | Median Survival Probability | Lower 95% | Upper 95% | Standard Error |
| --- | --- | --- | --- | --- | --- | --- | --- |
| Age=18-35, SEX=F | 3200 | 2998 | 201 | 0.9371 | 0.9288 | 0.9761 | 0.0028 |
| Age=18-35, SEX=M | 3901 | 3723 | 179 | 0.9541 | 0.9541 | 0.9476 | 0.0019 |
| Age=36-55, SEX=F | 12977 | 12088 | 896 | 0.9313 | 0.9269 | 0.9357 | 0.0027 |
| Age=36-55, SEX=M | 21086 | 19886 | 1213 | 0.9429 | 0.9398 | 0.9461 | 0.0021 |
| Age=56-64, SEX=F | 13385 | 12252 | 1143 | 0.9151 | 0.9104 | 0.9199 | 0.0038 |
| Age=56-64, SEX=M | 22683 | 21168 | 1533 | 0.9331 | 0.9298 | 0.9363 | 0.0034 |
| Age=65>, SEX=F | 6952 | 6130 | 829 | 0.8815 | 0.8739 | 0.8891 | 0.0044 |
| Age=65>, SEX=M | 11027 | 9988 | 1051 | 0.9053 | 0.8999 | 0.9108 | 0.0031 |

**Supplemental Table 3.** 180-day risk tables for pre- and post-MELD 3.0 groups stratified by sex.

| Strata | N | Number at Risk | Number of Events | Median Survival Probability | Lower 95% | Upper 95% | Standard Error |
| --- | --- | --- | --- | --- | --- | --- | --- |
| MELD-Na, SEX=F | 4363 | 4055 | 308 | 0.9294 | 0.9218 | 0.9370 | 0.0042 |
| MELD-Na, SEX=M | 7100 | 6666 | 434 | 0.9389 | 0.9333 | 0.9445 | 0.0030 |
| MELD 3.0, SEX=F | 6444 | 5945 | 499 | 0.9226 | 0.9161 | 0.9291 | 0.0036 |
| MELD 3.0, SEX=M | 9175 | 8615 | 560 | 0.9389 | 0.9341 | 0.9439 | 0.0027 |

**Supplemental Table 4.** Population differences across the two distinct MELD eras.


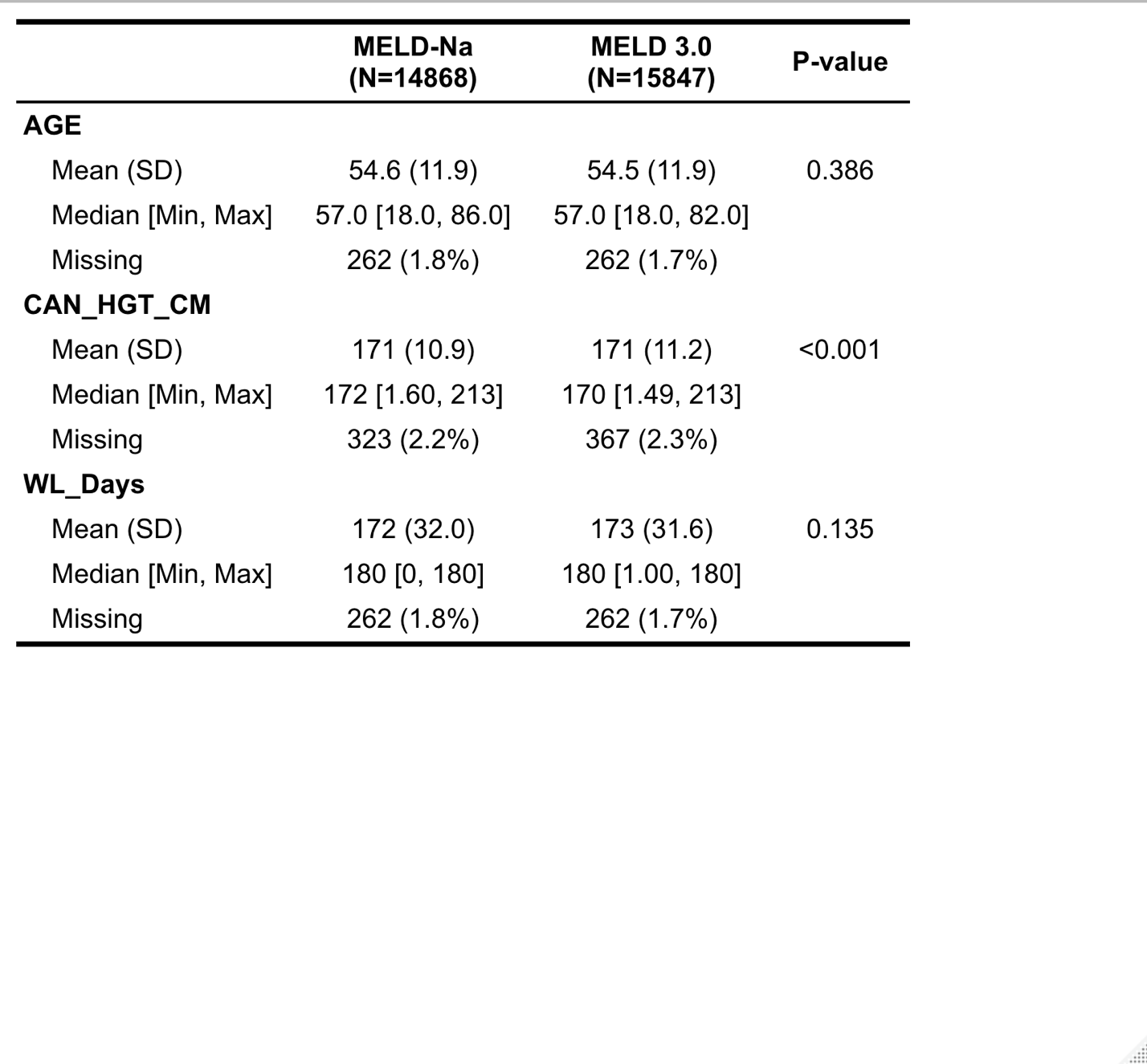


AGE = Age at listing; CAN_HGT_CM = Candidate height in centimeters at the time of listing
